# Supplementary material for: Dynalign II: common secondary structure prediction for RNA homologs with domain insertions
Source: Nucleic Acids Res. 2014 Nov 21;42(22):13939–48. doi: 10.1093/nar/gku1172 (PMC4267632; doi:10.1093/nar/gku1172)
Supplement: SUPPLEMENTARY DATA [file supp_gku1172_nar-02021-z-2014-File012.zip › manual/Text/ct2dot.html]

RNAstructure Command Line Help: ct2dot


|  |  |  |
| --- | --- | --- |
|  | RNAstructure Command Line Help ct2dot | - Contents - Index |
| ct2dot is used to generate a dot bracket file from a single structure within a CT file. The CT structure can be regenerated from the dot bracket structure by using the dot2ct interface. USAGE: ct2dot <ct file> <structure number> <bracket file> Required parameters: <ct file> \| The name of a CT file containing the structure to convert.; <structure number> \| The number, one-indexed, of the structure to convert in the CT file.; <bracket file> \| The name of a dot bracket file to which output will be written. Options which don't require added values: -h, -H, --help \| Display the usage details message. Options which require added values: NONE References: 1. Reuter, J.S. and Mathews, D.H. "RNAstructure: software for RNA secondary structure prediction and analysis." *BMC Bioinformatics*, 11:129. (2010). | | |
| Visit The Mathews Lab RNAstructure Page for updates and latest information. | | |
